# Arl8 and SKIP Act Together to Link Lysosomes to Kinesin-1

**DOI:** 10.1016/j.devcel.2011.10.007

**Published:** 2011-12-13

**Authors:** Cláudia Rosa-Ferreira, Sean Munro

**Affiliations:** 1 MRC Laboratory of Molecular Biology, Hills Road, Cambridge CB2 0QH, UK

## Abstract

Lysosomes move bidirectionally on microtubules, and this motility can be stimulated by overexpression of the small GTPase Arl8. By using affinity chromatography, we find that Arl8-GTP binds to the soluble protein SKIP (SifA and kinesin-interacting protein, aka PLEKHM2). SKIP was originally identified as a target of the *Salmonella* effector protein SifA and found to bind the light chain of kinesin-1 to activate the motor on the bacteria's replicative vacuole. We show that in uninfected cells both Arl8 and SKIP are required for lysosomes to distribute away from the microtubule-organizing center. We identify two kinesin light chain binding motifs in SKIP that are required for lysosomes to accumulate kinesin-1 and redistribute to the cell periphery. Thus, Arl8 binding to SKIP provides a link from lysosomal membranes to plus-end-directed motility. A splice variant of SKIP that lacks a light chain binding motif does not stimulate movement, suggesting fine-tuning by alternative splicing.

## Graphical abstract

**Graphical Abstract:**

**Figure:**
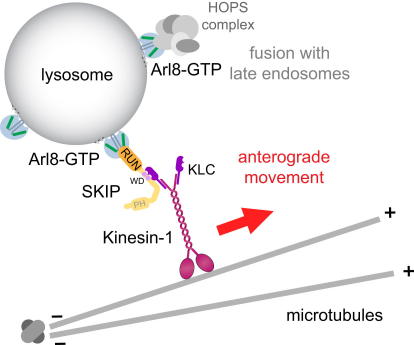

**Highlights:**

► The lysosomal GTPase Arl8 binds to the kinesin-1 linker SKIP ► SKIP and Arl8 are required for the normal intracellular distribution of lysosomes ► SKIP and Arl8 are required for the acid-induced centripetal movement of lysosomes ► SKIP contains a kinesin light chain binding site subject to alternative splicing

## Introduction

Lysosomes serve a wide range of roles in the turnover and processing of cellular and endocytosed material (Saftig and Klumperman, 2009). Like other organelles they move in both directions along microtubules to maintain their intracellular distribution and patrol the cytoplasm to ensure efficient function (Akhmanova and Hammer, 2010; Holzbaur and Goldman, 2010). In addition, it has been observed that outward movement of lysosomes is dramatically stimulated when cytoplasmic pH is lowered (Heuser, 1989). This has been suggested to be important for the response of cells to acidic microenvironments including those found in tumors (Cardone et al., 2005; Steffan et al., 2010). In knockout mice lacking the heavy chain of the major cytoplasmic kinesin, kinesin-1, lysosomes cluster around the microtubule-organizing center and the response to cytoplasmic acidification is impaired (Tanaka et al., 1998). This suggests that kinesin-1 is responsible for the anterograde movement of lysosomes, raising the question of how the motor is recruited to the lysosomal surface.

The mechanisms by which molecular motors are recruited to organelles are still not well understood, but in some cases linker proteins have been found which connect a particular motor to a small G protein on the surface of the organelle (Akhmanova and Hammer, 2010; Hirokawa and Noda, 2008). To date, the only small G protein reported to be present on mature lysosomes is the Arf-like G protein Arl8. Arl8 is conserved from humans to plants and protozoa, although it is absent from some yeasts and fungi, and there are two closely related paralogs in vertebrates, Arl8a and Arl8b. The human proteins are 91% identical and both of them, and the single Arl8 proteins in *Drosophila* and *Caenorhabditis elegans*, have been found to be on lysosomes (Bagshaw et al., 2006; Hofmann and Munro, 2006; Nakae et al., 2010). A *C. elegans* mutant lacking Arl8 shows defects in traffic between late endosomes and lysosomes, and also defects in the movement of presynaptic cargo along axons (Klassen et al., 2010). Overexpression in mammalian cells of either Arl8a or Arl8b stimulates the motility of lysosomes, resulting in them becoming more disperse and accumulating at the periphery of the cell (Bagshaw et al., 2006; Hofmann and Munro, 2006). This suggests that Arl8 recruits multiple effectors to lysosomes including those involved in both traffic and motility. In this paper, we report the result of a search for Arl8 effectors using affinity chromatography and, in particular, the finding that Arl8 binds to SKIP, a protein previously shown to act as a linker to kinesin-1 during *Salmonella* infection (Boucrot et al., 2005). SKIP has been shown to bind directly to kinesin light chain, the cargo-binding adaptor of kinesin-1, and this has been shown to cause kinesin to pull out long tubules or *Salmonella*-induced filaments (Sifs) from the *Salmonella*-containing vacuole (Boucrot et al., 2005; Dumont et al., 2010). Moreover, it has been noted that when levels of SKIP are increased or reduced in uninfected cells then the localization of the Golgi and lysosomes is affected (Dumont et al., 2010). Our finding that Arl8 is responsible for attaching SKIP to lysosomes provides an effector for Arl8 and establishes a complete chain of connection from lysosomal membrane to motor.

## Results

### SKIP Is an Effector for Arl8

**Figure 1:**
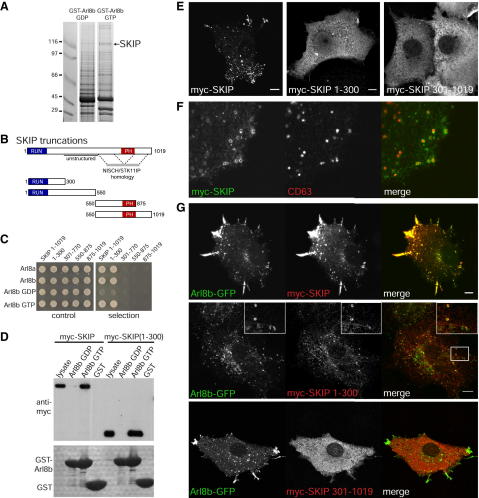
Arl8 Binds to the RUN Domain of SKIP, and the Two Proteins Colocalize on Lysosomes (A) Coomassie-stained protein gel of eluates from large-scale affinity chromatography of HeLa cytosol using GST fusions to Arl8b-T34N (GDP) or Arl8b-Q75L (GTP). Each lane represents 1.6 × 10^8^ cells. Bands associated with Arl8b-Q75L were excised and mass spectrometry of tryptic peptides identified seven sequences corresponding to SKIP. Other bands examined were heat shock proteins or abundant cytosolic enzymes. (B) Schematic of the organization of SKIP indicating the RUN and PH domains, and the region predicted to be unstructured. The regions flanking the PH domain are distantly related to a region of unknown function found at the C-termini of nischarin and STK11IP (serine/threonine kinase 11 interacting protein) (Lim and Hong, 2004). (C) Yeast two-hybrid interactions between Arl8a or Arl8b, or the latter with T34N (GDP) or Q75L (GTP) mutations, and SKIP or the indicated truncations. (D) Immunoblot of the eluates from affinity chromatography of COS cell cytosol using GST-fusions to Arl8b as in (A). The cells were transiently expressing full-length SKIP or SKIP(1-300) with N-terminal myc tags. The lower panel is a Coomassie-stained gel of the total material on the glutathione-Sepharose beads to indicate the levels of GST fusion proteins. (E) Confocal micrographs of COS cells expressing the indicated versions of SKIP and stained for the myc epitope. Scale bars 10 μm. (F) Confocal micrographs of the periphery of a COS cell expressing the full-length SKIP and costained for the lysosomal marker CD63. (G) Confocal micrographs of COS cells cotransfected with plasmids expressing Arl8b-GFP and the indicated versions of SKIP. For SKIP(1-300) a region is enlarged in the inset panel to show the colocalization and the tubes that are particularly prominent with this truncation, although were also sometimes observed with the full-length protein. Scale bars 10 μm. See also Figure S1.

To identify effectors for Arl8, we performed affinity chromatography with immobilized human Arl8b fused to GST. The protein was prepared with mutations that lock other Arf family GTPases in the GTP- or GDP-bound states, with these mutations being known to confer constitutive activity, or loss of activity, respectively, to Arl8b in vivo (Bagshaw et al., 2006; Hofmann and Munro, 2006). Cytosol from HeLa cells was incubated with the GST fusions immobilized on beads and after washing, the bound proteins eluted. A protein migrating at approximately 150 kDa bound specifically to Arl8b-GTP, and mass spectrometry of tryptic peptides identified it as SKIP, the product of the human gene PLEKHM2 (Figure 1A). SKIP was originally identified as binding to the SifA protein that is secreted by *Salmonella* from its replicative vacuole, and it is proposed to bind and activate kinesin-1 on the replicative vacuole and thus induce the formation of *Salmonella*-induced filaments (Boucrot et al., 2005; Diacovich et al., 2009; Jackson et al., 2008; Ohlson et al., 2008). SKIP is a soluble cytosolic protein of 1019 residues that has a RUN domain and a PH domain separated by a less well-conserved region that is predicted to be an unstructured linker (Figure 1B). Our PSI-BLAST searches revealed that the PH domain is flanked by conserved regions that are both distantly related to a region found in two proteins of unknown function, nischarin and STK11IP, the former of which has been found on endosomes (Lim and Hong, 2004). This suggests the C-terminal half of the protein may comprise a large globular domain into which the PH domain is embedded.

The ability of Arl8 to bind SKIP was confirmed using both yeast two-hybrid assays and affinity chromatography of epitope-tagged SKIP expressed in COS cells. In both cases, the binding was only to the GTP form of Arl8, and in the yeast two-hybrid assay both Arl8a and Arl8b showed an interaction (Figures 1C and 1D). To map the Arl8 binding site on SKIP, we used the yeast two-hybrid assay to test interactions with truncated versions of the protein. The N-terminal 300 residues containing the RUN domain were necessary and sufficient for GTP-dependent binding to Arl8 as determined by yeast two-hybrid assay and affinity chromatography (Figures 1C and 1D).

### Arl8b Recruits SKIP to Lysosomes

We next compared the intracellular distribution of Arl8b and SKIP in tissue culture cells. SKIP was originally reported to have a diffuse distribution when expressed in cells (Boucrot et al., 2005), but we found that at moderate expression levels the full-length protein was punctate, and these puncta colocalized with the lysosomal marker CD63 (Figures 1E and 1F), and a recent report agrees with this localization (Dumont et al., 2010). Overexpression of SKIP caused the lysosomal compartment to accumulate at the periphery of the cell (Figure 1E). When we coexpressed SKIP with Arl8b, the two proteins colocalized and the peripheral displacement of lysosomes was enhanced (Figure 1G).

The truncation SKIP1-300, which binds to Arl8, showed surprisingly a diffuse distribution in cells, with a similar distribution also seen for the remainder of the protein (SKIP301-1019, Figure 1E). However, coexpression of Arl8b caused SKIP1-300 to accumulate on lysosomes, but had no effect on SKIP301-1019 (Figure 1G). This indicates that Arl8b binds to the N terminus of SKIP in vivo, but suggests that at physiological levels of Arl8 further interactions mediated via the C-terminal domain of SKIP are needed for its stable membrane association. In addition, when SKIP1-300 was coexpressed with Arl8, then both proteins were seen to decorate long tubules similar to the *Salmonella*-induced filaments that require SKIP for their formation in *Salmonella*-infected cells (Figure 1G). One possible explanation is that SKIP1-300 does not associate as tightly with lysosomes as the full-length protein and that this results in the pulling out of membrane tubules rather than the whole scale relocalization of the organelle.

In cells overexpressing SKIP there was also a perturbation of the Golgi apparatus (see Figure S1A available online). We quantified the degree of overexpression by protein blotting of cell populations, which were also imaged to determine transfection efficiency and organelle perturbation. When cells expressing SKIP or Arl8b were divided into sets with low, medium, and high expression, we found that displacement of lysosomes by SKIP was widespread even in the low overexpression set (average 3.1-fold overexpression), whereas Golgi perturbation was only widespread in the higher expressing sets and not seen with Arl8b (Figure S1D). The ER or early endosomes, other organelles that move on microtubules, were unperturbed (Figure S1D). Residues 1–300 of SKIP are necessary and sufficient for the effect on the Golgi (Figure S1B), consistent with a recent report (Dumont et al., 2010). It is possible that this effect is due to SKIP directly recruiting kinesin-1 to Golgi membranes, although we have not been able to detect either Arl8 or SKIP on the Golgi (Figure S1C; Hofmann and Munro, 2006). Alternatively, SKIP overexpression may titrate out motor regulators to allow plus-end-directed forces on the Golgi to overcome those that normally drive it toward minus ends.

### Arl8 and SKIP Act Together in Plus-End-Directed Motility of Lysosomes

**Figure 2:**
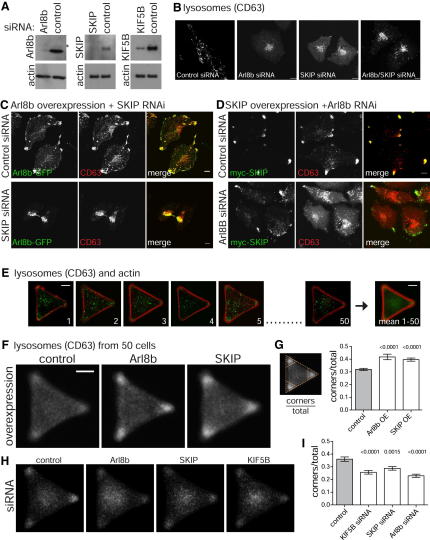
SKIP and Arl8 Are Required for Accumulation of Lysosomes at Microtubule Plus Ends (A) Immunoblots of cell lysates from HeLa cells treated with siRNAs against the indicated targets. The blots were probed to assess levels of endogenous Arl8a and Arl8b, endogenous SKIP or endogenous KIF5B. The anti-Arl8b antisera also recognizes Arl8a which is a faint band migrating slower than Arl8b (asterisk). (B) Confocal micrographs of HeLa cells treated with the indicated siRNAs and stained for CD63 to label lysosomes. Scale bars 10 μm. (C) Confocal micrographs of HeLa cells expressing Arl8b-GFP treated with siRNA to knockdown SKIP, and then labeled for CD63. The peripheral accumulation of CD63 induced by Arl8b overexpression is prevented by removal of SKIP. Scale bars 10 μm (D) Confocal micrographs of HeLa cells expressing myc-SKIP treated with siRNA to knockdown Arl8b, and then labeled for CD63. Removal of Arl8b prevents SKIP inducing the peripheral accumulation of lysosomes. Scale bars 10 μm. (E) Confocal micrographs of individual HeLa cells plated on a micropatterned array (Y shape, small; CYTOO Inc.) and stained with anti-CD63 (lysosomes) and phalloidin (actin), along with an average image obtained from superimposing 50 individual cells. (F) Average distribution of lysosomal staining from sets of 50 cells analyzed as in (E), either mock-transfected or with overexpression of Arl8b-GFP or myc-SKIP as indicated. Scale bar 10 μm. (G) Quantitation of lysosomal staining in the corners of the cells as ratio to the total lysosomal staining. Corners were defined by three triangles each being one-ninth the area of that used to outline the whole cell, and the corner/total ratio determined for 50 cells (error bars show standard error of mean). For both proteins, the difference to the control is statistically significant (unpaired two-tailed t test). (H) Average distribution of lysosomal staining from sets of 50 cells as in (F), but following treatment with siRNAs against the indicated genes. (I) Quantitation of lysosomal staining as in (G). 50 cells were measured for each set and in each case the difference to the control is statistically significant (unpaired two-tailed t test). See also Figure S2.

The above results show that Arl8b and SKIP overexpression is sufficient to pull lysosomes toward the plus ends of microtubules. However, the key issue is whether these proteins are actually necessary for lysosomal movement at native levels. To address this, we used siRNAs to knockdown the levels of the proteins. We raised antibodies against Arl8b that recognize both Arl8a and Arl8b. The latter protein appears to predominate in HeLa cells, consistent with its mRNA being expressed ubiquitously while expression of Arl8a shows a more restricted tissue distribution (Kwon et al., 2009; Su et al., 2004). SiRNAs against Arl8b reduced total Arl8 levels by more than 95%, and we were able to achieve a knockdown of 80% of SKIP (Figure 2A). Knockdown of SKIP resulted in an accumulation of lysosomes in the perinuclear region consistent with the effect reported previously (Jackson et al., 2008). Knockdown of Arl8 showed a similar effect, indicating that both proteins are required for lysosomes to move away from the microtubule-organizing center and toward the plus ends of microtubules (Figure 2B).

We next examined whether each protein is required for the effects seen when the other is overexpressed. In cells overexpressing Arl8b, knockdown of SKIP prevented the peripheral accumulation of lysosomes (Figure 2C). This indicates that most, if not all, of the stimulation of plus-end-directed movement by Arl8 requires SKIP. Likewise in cells overexpressing SKIP, knockdown of Arl8 resulted in lysosomes remaining throughout the cytosol instead of accumulating at the cell periphery (Figure 2D). In contrast knockdown of Arl8 did not prevent the perturbation of the Golgi induced by SKIP overexpression (Figure S2A). This suggests that if SKIP is acting directly on Golgi membranes, then it is not being recruited by Arl8. In addition, knockdown of SKIP did not induce fragmentation of the Golgi ribbon and markers of different cisternae remained discrete (Figure S2B), suggesting again that SKIP may not act on the Golgi apparatus when present at endogenous levels. Finally, other organelles that move on microtubules did not show relocation in SKIP siRNA treated cells (ER, mitochondria, peroxisomes and early endosomes; n > 210 transfected cells). This indicates that even if SKIP has other roles, its primary site of action is on lysosomes.

### Quantification of the Effects of Altering the Levels of Arl8b and SKIP

Because fibroblast cells do not have a uniform shape the distribution of lysosomes varies somewhat between individual cells, especially when the lysosomes accumulate at the periphery. Thus to quantify the intracellular distribution of lysosomes we used micropatterned arrays to allow comparison of cells of a fixed size and shape. Use of a Y-shaped pattern results in triangular cells with lysosomes scattered throughout the cytoplasm (Figure 2E). We overlaid images of 50 cells for each condition and so determined the average distribution of lysosomes within the cells. We also determined the proportion of lysosomal staining that was in the periphery, ie the corners, for each cell of the set to allow quantification. This confirmed that increasing the levels of Arl8b or SKIP resulted in a movement of lysosomes toward the periphery that was statistically significant (Figures 2F and 2G). In contrast, when Arl8 and SKIP were knocked down by RNAi, the peripheral pool of lysosomes was reduced and again the effect was highly statistically significant (Figures 2H and 2I).

### Arl8b and SKIP Exert Their Effects via Kinesin Light Chain

**Figure 3:**
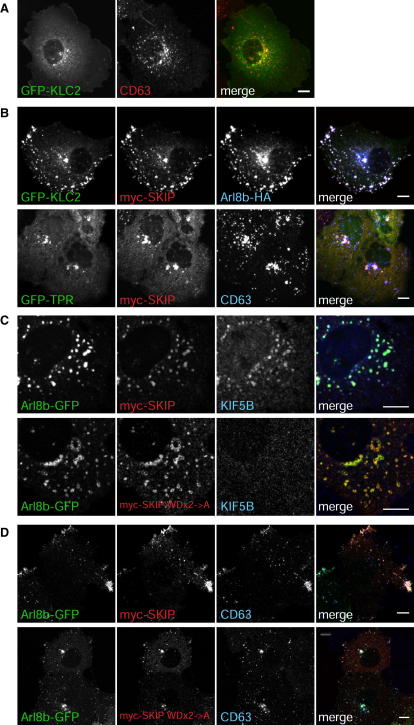
Arl8b and SKIP Recruit Kinesin-1 to Lysosomes (A) Confocal micrographs of COS cells expressing GFP-KLC2 and stained for CD63. (B) As in (A), except the cells were transfected with plasmids expressing the indicated proteins. (C) Confocal micrographs of the distribution of KIF5B in COS cells expressing GFP-Arl8b and either wild-type myc-SKIP or a mutant with both “WD” motifs replaced with alanines (W207A, D208A, W236A, E237A, “WDx2->A”). Cells were treated with nocodazole (10 μM, 3 hr) prior to fixation. (D) Confocal micrographs of the distribution of the lysosomal marker CD63 in COS cells expressing GFP-Arl8b and either myc-SKIP or the WDx2->A SKIP mutant. Scale bars 10 μm. See also Figure S3.

Previous analysis of SKIP has shown that it interacts with kinesin light chain, a protein that contains a TPR domain which typically binds kinesin cargos, and a coiled-coil region that interacts with kinesin heavy chain (Boucrot et al., 2005; Dumont et al., 2010). A fragment of SKIP comprising residues 1–310 binds to the TPR domain of kinesin light chain, and this interaction is responsible for linking SKIP to kinesin-1 heavy chain (Dumont et al., 2010). The predominant nonneuronal form of kinesin light chain is KLC2 (Rahman et al., 1998), and when we overexpressed GFP-tagged KLC2, most accumulated in the cytosol with relatively little accumulation on lysosomes (Figure 3A). However, when coexpressed with Arl8b and SKIP, GFP-KLC2 was almost entirely recruited to the outwardly spread lysosomes labeled by these two proteins (Figure 3B). In addition, endogenous KIF5B, the heavy chain of kinesin-1, accumulated on lysosomes in cells overexpressing Arl8b and SKIP (Figure 3C). The TPR domain of KLC2 binds SKIP and others cargos but does not link to KIF5B (Dumont et al., 2010; Rietdorf et al., 2001). When GFP-TPR was overexpressed with SKIP then, in contrast to GFP-KLC2, the two proteins were on lysosomes that clustered at the center of the cell (Figure 3B). Likewise, when KIF5B was knocked down by >85% by RNAi, lysosomes were more centrally clustered to a degree similar to that seen with knockdown of Arl8 or SKIP (Figures 2A, 2H, and 2I). Taken together, these results indicate that the ability of SKIP to bind kinesin light chain is not competed by association with Arl8b, and that the association between SKIP and kinesin light chain, and thus kinesin-1, is responsible for plus-end-directed movement mediated by SKIP.

### Alternatively Spliced Forms of SKIP Differ in Their Effect on Lysosome Distribution

**Figure 4:**
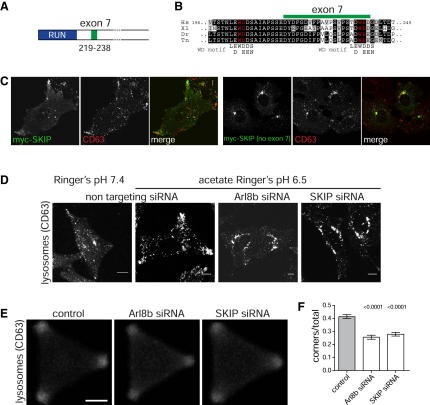
SKIP Contains a KLC2 Binding Motif and Is Required for Acidification-Induced Movement of Lysosomes (A and B) Schematic of human SKIP showing the region encoded by exon 7 which is skipped in some mRNAs, and an alignment with the same region of SKIP orthologs from other vertebrates (Xl, *Xenopus laevis*, Dr, *Danio rerio*, Tn, *Tetraodon nigroviridis*). The regions similar to the kinesin-binding WD motif are highlighted in red, with the motif shown underneath (Morgan et al., 2010). (C) Confocal micrographs showing the distribution of lysosomes in cells expressing the two splice variants of SKIP. The variant lacking exon 7 does not induce a peripheral accumulation of lysosomes. (D) Confocal micrographs of HeLa cells stained for the lysosomal marker CD63 with, or without, treatment with acetate Ringer's solution pH 6.5 for 30 min to acidify the cytosol. Lysosomal movement to the periphery was reduced by RNAi of Arl8b or SKIP. (E and F) Quantitation of siRNA-treated cells on a micropatterned array as in Figure 2F shows reduced peripheral movement even after prolonged acetate treatment (60 min) See also Figure S4.

When we searched the expressed sequence tag (EST) databases with human SKIP, we found some ESTs that lacked a short region within the N-terminal region. This reflects the variable inclusion of exon 7 of the human SKIP gene, and results in the presence or absence of residues 219–238 of the longer variant (Figures 4A and 4B). ESTs from other species revealed that this alternative splicing is conserved in SKIP orthologs from birds, fish, and frogs, indicating that it has biological value. PCR on cDNA from a panel of human tissues revealed that the two splice forms are widely expressed, with the longer form being ubiquitous and the shorter form being present in many but not all tissues at variable levels (Figure S4).

Interestingly, the region affected by alternative splicing contains a conserved region that is a close match to the consensus for a kinesin light chain binding site recently found in several proteins including calsyntenin-1/alcadein, caytaxin, and vaccinia virus A36 (Figure 4B; Konecna et al., 2006; Morgan et al., 2010). These “WD” motifs often occur in a pair, and in SKIP a second such WD motif is present N-terminal to that in the optional exon (Figure 4B). Overexpression of the shorter variant of SKIP does not result in accumulation of lysosomes at the periphery, but in contrast causes accumulation in the perinuclear region (Figure 4C). Taken together, these results suggest that the ability of SKIP to stimulate outward movement of lysosomes is controlled by alternative splicing and hence the ratio of the two SKIP splice variants could provide a mechanism to fine-tune the distribution of lysosomes in different cell types.

To further investigate the importance of these WD motifs for recruiting kinesin, we mutated them both in the context of the long form of SKIP that contains exon 7. When coexpressed with Arl8b, this mutant form of SKIP (W207A, D208A, W236A, E237A; “WDx2->A”) accumulated on lysosomes just like wild-type SKIP (Figure S3). However, the lysosomal accumulation of KIF5B was abolished by the WD motif mutations, confirming that the WD motifs link SKIP to kinesin-1 (Figure 3C). This mutant form of SKIP did not induce a peripheral scattering of lysosomes with instead an accumulation in the perinuclear region (Figure 3D). This demonstrates that the movement of lysosomes to the periphery induced by Arl8b and SKIP is dependent on the ability of the latter protein to bind kinesin-1.

### SKIP and Arl8b Are Required for Cytoplasmic pH Control of Lysosome Location

The position of lysosomes within a cell is known to be regulated by cytoplasmic pH, with a reduction in pH inducing outward movement and accumulation of lysosomes at the cell periphery (Heuser, 1989). This outward movement is dependent on the kinesin-1 heavy chain, but what machinery recruits the motor for the movement is unknown (Tanaka et al., 1998). We thus examined the effect of reducing cytoplasmic Arl8b and SKIP using siRNA. Figure 4D shows that knockdown of either protein inhibits the acidification-dependent outward movement. Quantitation of cells on a patterned microarray confirmed a clear reduction in the acid-induced peripheral pool even after prolonged exposure to acidifying conditions (Figures 4E and 4F).

## Discussion

The observations reported here identify SKIP as an effector for Arl8 and show that SKIP is required for Arl8 to exert its effects on lysosome location. We find that SKIP contains kinesin light chain binding WD motifs, and these are required for kinesin-1 recruitment and the peripheral movement of lysosomes induced by Arl8b and SKIP overexpression. This indicates that Arl8 and SKIP act together to recruit kinesin-1 to lysosomes and hence direct their movement toward microtubule plus ends. Previous studies of knockout mice have found that removal of KIF5B, the ubiquitous (nonneuronal) form of kinesin-1 heavy chain results in lysosomes redistributing to the center of the cell (Tanaka et al., 1998). However, this need not preclude other kinesins having a role in lysosomal motility in specialized cell types or in particular circumstances (Brown et al., 2005; Matsushita et al., 2004; Teng et al., 2005). We did not observe KIF3B (kinesin-2) being recruited to lysosomes in cells overexpressing Arl8b and SKIP, and a motorless form of KIF1Bβ (kinesin-3) did not prevent the peripheral redistribution of lysosomes by overexpressed Arl8b and SKIP (data not shown). Thus, if other kinesins contribute to lysosome motility they may use different adaptors. Minus-end-directed movement of lysosomes requires dynein (Harada et al., 1998), but we did not observe any lysosomal accumulation of dynein heavy chain in cells overexpressing Arl8b and SKIP (data not shown).

Our results provide strong evidence that Arl8 recruits kinesin-1 to lysosomes by binding to SKIP that then binds via its WD motifs to kinesin light chain. However it is likely that both Arl8 and SKIP have additional roles in the cell. Arl8 is the only GTPase known to be constitutively present on mature lysosomes and so it is likely to recruit other effectors including those involved in membrane traffic (Nakae et al., 2010). Indeed, mammalian Arl8b has recently been reported to bind to the Vps41 subunit of the HOPS complex, which provides an explanation for its Arl8's additional role in traffic to lysosomes (Garg et al., 2011). Likewise the C-terminal region of SKIP could contribute to motor regulation, or have a distinct function in membrane traffic. Like other organelles, lysosomes require a careful balance of plus-end- versus minus-end-directed motility, and so motor recruitment is likely to be complex and tightly regulated (Akhmanova and Hammer, 2010; Holzbaur and Goldman, 2010). Indeed, recruitment of kinesin-1 to the *Salmonella*-containing vacuole requires a second factor PipB2 that binds directly to kinesin, and the precise relationship between SifA and PipB2 remains unclear (Dumont et al., 2010; Henry et al., 2006). Efforts to examine the role of Arl8 and SKIP in lysosomal membrane trafficking processes are hampered by the fact that changing their levels affects lysosome location, which could indirectly affect lysosomal function. Progress on this issue is likely to require identification of further binding partners for both proteins.

Nonetheless it is clear that Arl8 and SKIP constitute a core link between lysosomal membranes and kinesin-1. This link appears to be required for maintaining the intracellular distribution of lysosomes, and for the outward movement of lysosomes in response to cytosolic acidification. This pH control of lysosome motility has been proposed to be important for cellular behavior in acidic microenvironments and, in particular, to contribute to metastasis and tumor invasion due to release of lysosomal proteases (Cardone et al., 2005; Steffan et al., 2010). Identification of Arl8 regulators could thus provide a route to pharmacological control of these processes.

## Experimental Procedures

### Cell Culture and RNAi

HeLa and COS-7 cells were grown in DMEM/10% fetal calf serum and transfected with FuGene (Roche). Cell were fixed in 4% formaldehyde 24–48 hr posttransfection, blocked, and permeabilized with PBS, 0.5% Triton X-100, 20% FCS for 1 hr and labeled with antibodies in the same solution. Images were obtained with a Zeiss LSM 710 confocal microscope. Plasmids expressing GFP-KLC2 and TPR were from Michael Way (Rietdorf et al., 2001).

TARGETplus siRNAs were used according to manufacturer's instructions (Dharmacon). In brief, cells were transfected with 50 nM of siRNA twice (interval of 24 hr) with Oligofectamine (Invitrogen) and analyzed around 54 hr after first transfection. When necessary, cells were transfected with plasmid DNA and FuGene 6 hr after the first siRNA transfection. SiRNAs were 5′-GAUAGAAGCUUCCCGAAAU-3′ for ARL8B; 5′-CUUCUGAACUGGACCGAUU-3′ for SKIP; ON-TARGETplus SMART pool for KIF5B (Dharmacon). Nontargeting siRNA #1 (Dharmacon) was used for all negative controls.

### Affinity Chromatography of Arl8b Effectors

GST- Arl8b T34N and GST- Arl8b Q75L were produced in *Escherichia coli* strain BL21-GOLD (DE3) and used for affinity chromatography of cytosol as described previously (Sinka et al., 2008). Large-scale affinity chromatography was performed with pellets from 0.5 l bacterial cultures and 1 × 10^9^ HeLa cells. Small-scale binding was with a 10 cm^2^ dish of transfected COS-7 cells and 0.5 g of bacterial pellet.

### Yeast-Two Hybrid Assays

Bait plasmids (Arl8b T34N and Arl8b Q75L lacking the first 17 residues) were cloned into pDEST32 (Invitrogen) and transfected into the yeast strain PJ69-4a (James et al., 1996). Prey fragments were cloned into pDEST22 (Invitrogen) and transfected into the yeast strain PJ69-4α. Strains were mated, grown for 24 hr in YEPD, and replicates grown on selective medium at 30°C for 3 days.

### Antibodies and Immunodetection

Rabbit antisera to GST-tagged Arl8b without the N-terminal helix (Δ1-17 amino acids) were raised using a 28 day protocol (Eurogentec). The serum was precleared with GST followed by affinity chromatography using the antigen. Rabbit anti myc and HA epitope tags (Santa Cruz Biotechnology), anti-calreticulin (208910, Calbiochem/Merck), anti-TGN46 (T7576, Sigma-Aldrich), anti-giantin (HPA011008, Sigma-Aldrich) and anti-SKIP (Boucrot et al., 2005); goat anti-KIF5B (ab15705, AbCam); and mouse monoclonals anti-CD63 (H5C6, Developmental Studies Hybridoma Bank), anti-EEA1 (610457, BD Biosciences), anti-ERGIC-53 (G19/3, Enzo Life Sciences), anti-dynein (74.1, Abcam) and anti-GM130 (610823, BD Biosciences), were detected by secondary antibodies conjugated with Alexa fluorochromes (Invitrogen). For protein blots, secondary antibodies conjugated with HRP (Dako) were detected by chemiluminescence.

## Supplemental Information

Document S1. Four Figures
