# Supplementary material for: Arl8 and SKIP Act Together to Link Lysosomes to Kinesin-1
Source: Dev Cell. 2011 Dec 13;21(6):1171–8. doi: 10.1016/j.devcel.2011.10.007 (PMC3240744; doi:10.1016/j.devcel.2011.10.007)
Supplement: Document S1. Four Figures [file mmc1.pdf]

Developmental Cell, Volume *21*

Supplemental Information

Arl8 and SKIP Act Together to Link Lysosomes to Kinesin-1

Cláudia Rosa-Ferreira and Sean Munro

Supplemental Inventory

Figure S1. Fragmentation of the Golgi in cells overexpressing SKIP (related to Figure 1)

Figure S2. Effect of Arl8 and SKIP knockdown on the Golgi apparatus (related to Figure 2)

Figure S3. The kinesin light chain binding site of SKIP is not required for lysosomal targeting (related to Figure 3)

Figure S4. Tissue distribution of the inclusion of exon 7 in SKIP transcripts (related to Figure 4)

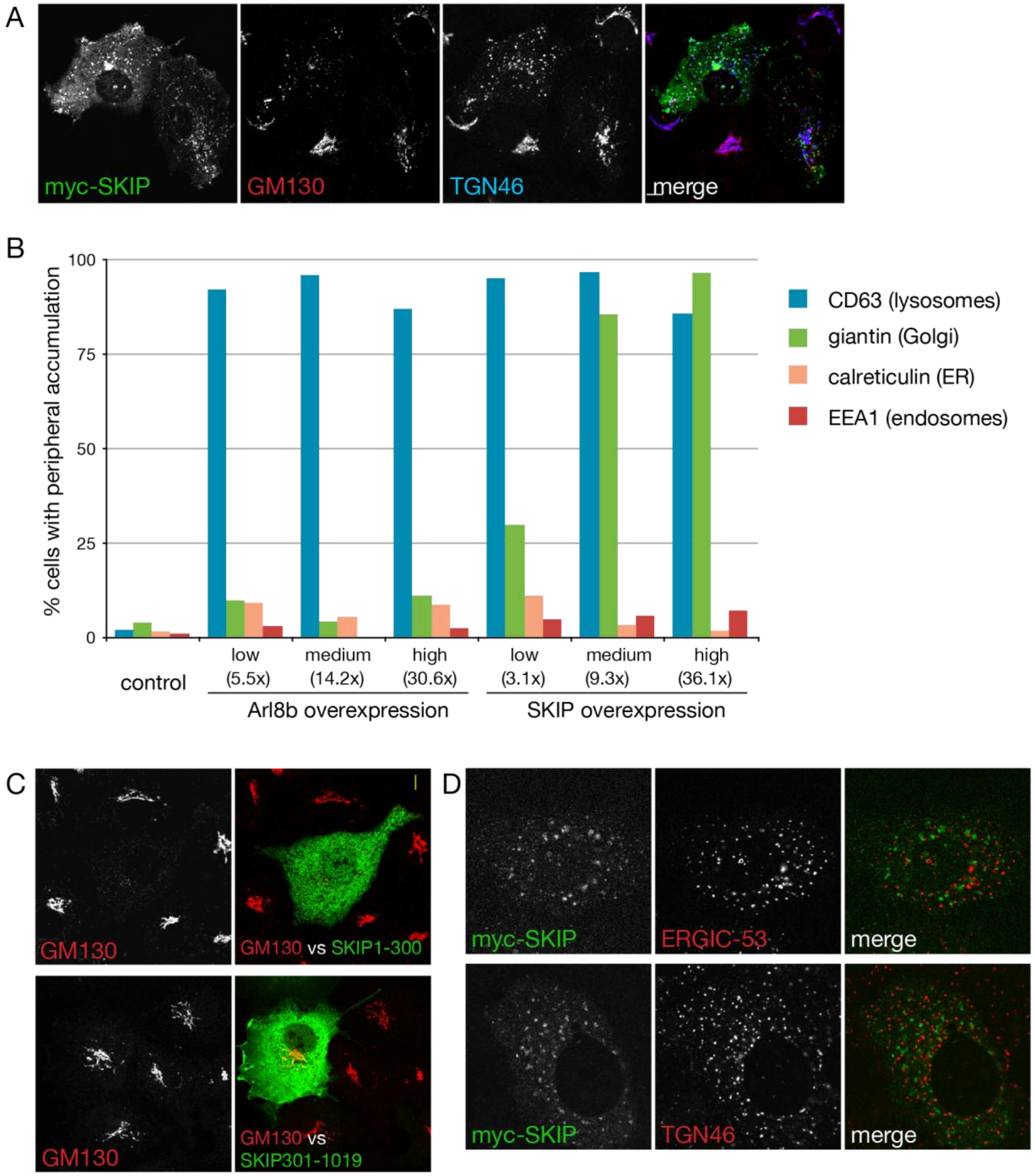

Figure S1. Fragmentation of the Golgi in cells overexpressing SKIP (related to Figure 1) (A) Confocal micrographs of COS cells expressing SKIP and co-stained for Golgi markers. Expression of SKIP causes the Golgi to be scattered through the cytoplasm and fragmented.

(B) Quantitation of organelle relocation in cells overexpressing GFP-Arl8b or myc-SKIP. Transfected COS cells were stained for the indicated organelle markers and scored for those where the organelle accumulated, at least in part, in the cell periphery. The cells were divided into low, medium and high categories depending on expression levels (n=30-50 depending on category). The level of the overexpressed proteins per cell was quantified from confocal images, and the total cell extract from the transfected population probed by immunoblotting with antibodies to Arl8b or SKIP to determine the average level of overexpression in the population. This was converted to a mean level of overexpression per category, and this is indicated. Arl8b overexpression only relocates lysosomes, whereas SKIP strongly affects lysosomes even at the lowest level, with higher levels perturbing the Golgi, but not other microtubule dependent organelles.

(C) as (A), except COS cells are expressing the indicated SKIP truncations. SKIP(1-300), but not SKIP(301-1019), scatters the Golgi.

(D) Confocal micrographs of COS cells showing the distribution of transfected myc-SKIP along with cis-Golgi (ERGIC-53) or trans-Golgi (TGN46) markers. The cells were treated with nocodazole (10 μM, 3 hours) prior to fixation, and myc-SKIP does not detectably accumulate on the resulting Golgi mini-stacks.

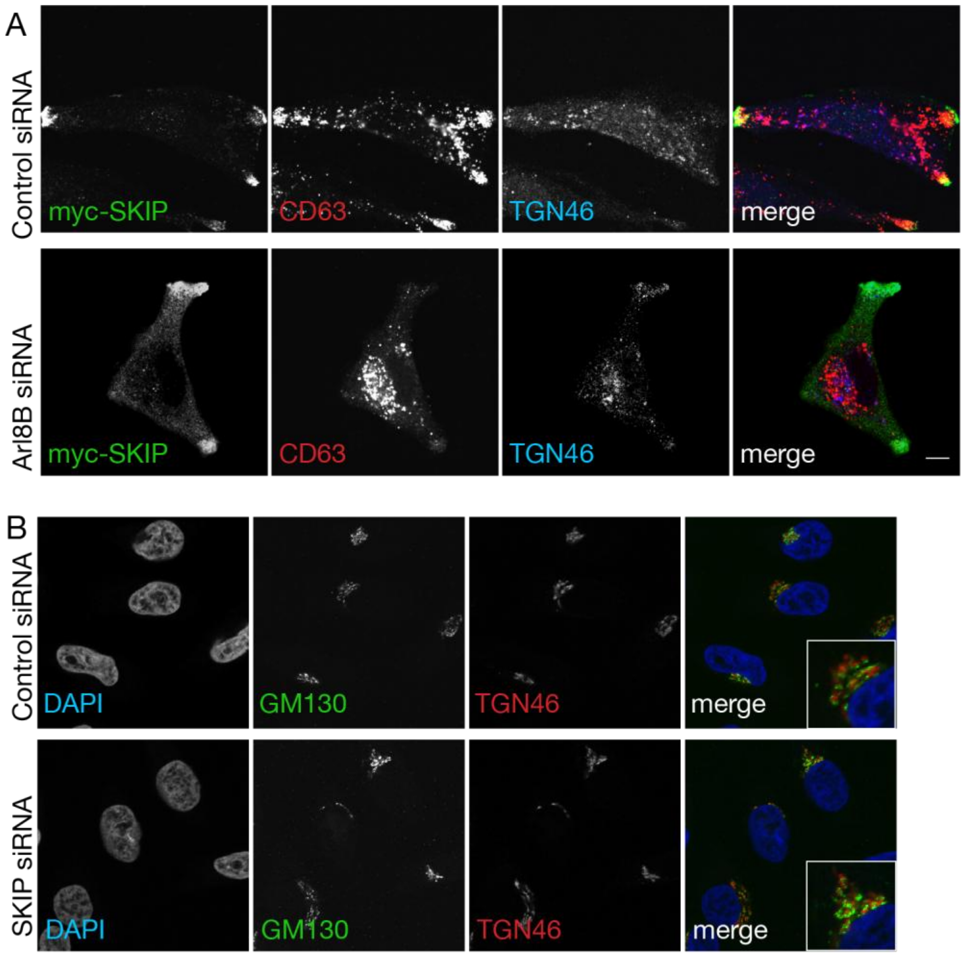

Figure S2. Effect of Arl8 and SKIP knockdown on the Golgi apparatus (related to Figure 2)

(A) Confocal micrographs of HeLa cells expressing myc-SKIP treated with siRNA to knock down Arl8b, and then labelled for CD63 and the Golgi marker TGN46. Removal of Arl8b prevents SKIP inducing the peripheral accumulation of lysosomes, but does not prevent the fragmentation of the Golgi apparatus.

(B) Confocal micrographs of HeLa cells treated with siRNA to knockdown SKIP and labelled for the markers for the cis-Golgi (GM130) and trans-Golgi (TGN46). The Golgi ribbon does not detectably fragment, and the cisternal-specific markers remain discrete (inset).

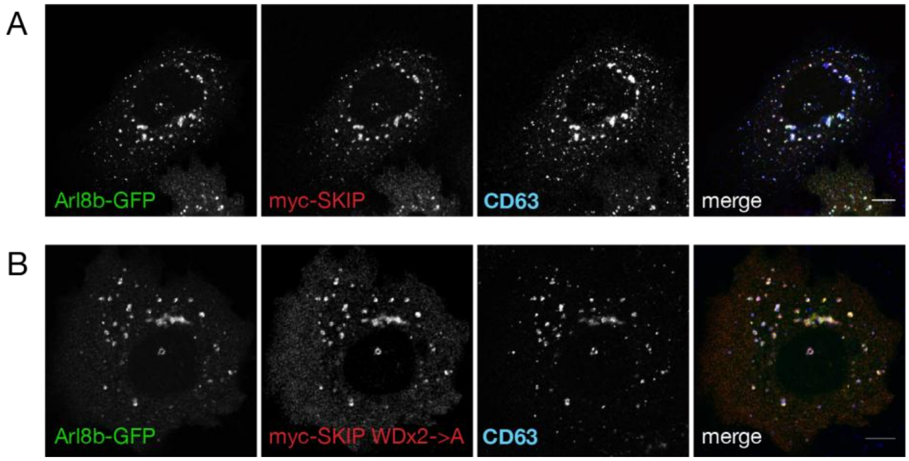

Figure S3. The kinesin light chain binding site of SKIP is not required for lysosomal targeting (related to Figure 3)

(A) Confocal micrographs of COS cells co-expressing myc-SKIP and GFP-Arl8b and co-stained for the lysosomal marker CD63. Cells were treated with nocodazole (10 μM, 3 hours) prior to fixation.

(B) as in (A), except SKIP is mutated to replace the two kinesin light chain binding motifs (WD and WE) with two alanines. The mutant form still accumulates on lysosomes like the wild-type protein.

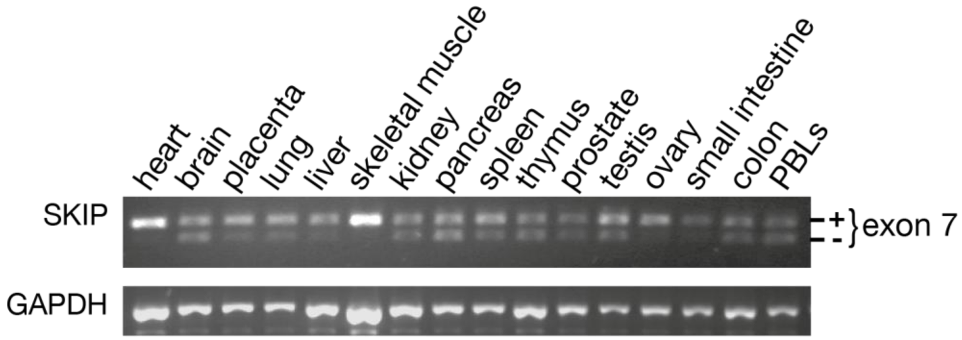

Figure S4. Tissue distribution of the inclusion of exon 7 in SKIP transcripts (related to Figure 4)

PCR products amplified with primers for SKIP or for the control GAPDH from a multiple tissue cDNA panel (Clontech; PBLs, peripheral blood leucocytes). The SKIP primers (CTTACCTAGACCTGGCCCCCTACATG and GTCTCTGCCGGCTCCTCGTTGAAG) flank a region containing exon 7 and so the two bands correspond to mRNA with, or without, this exon as indicated.
